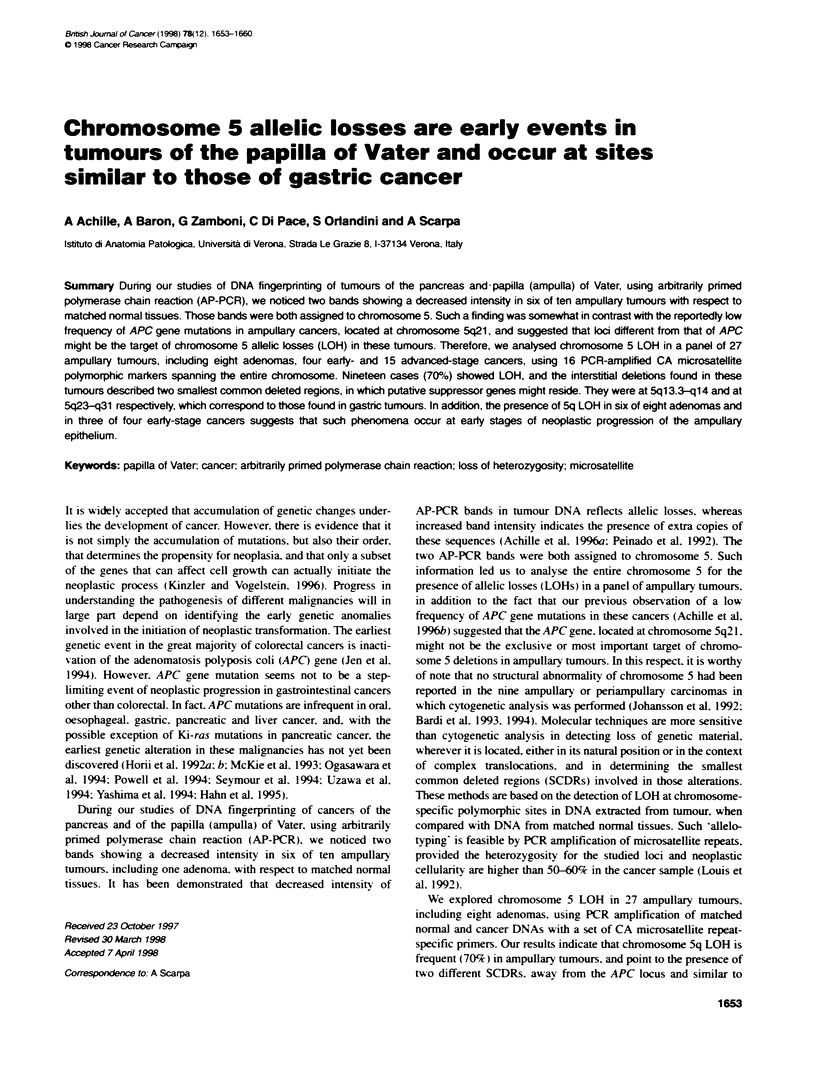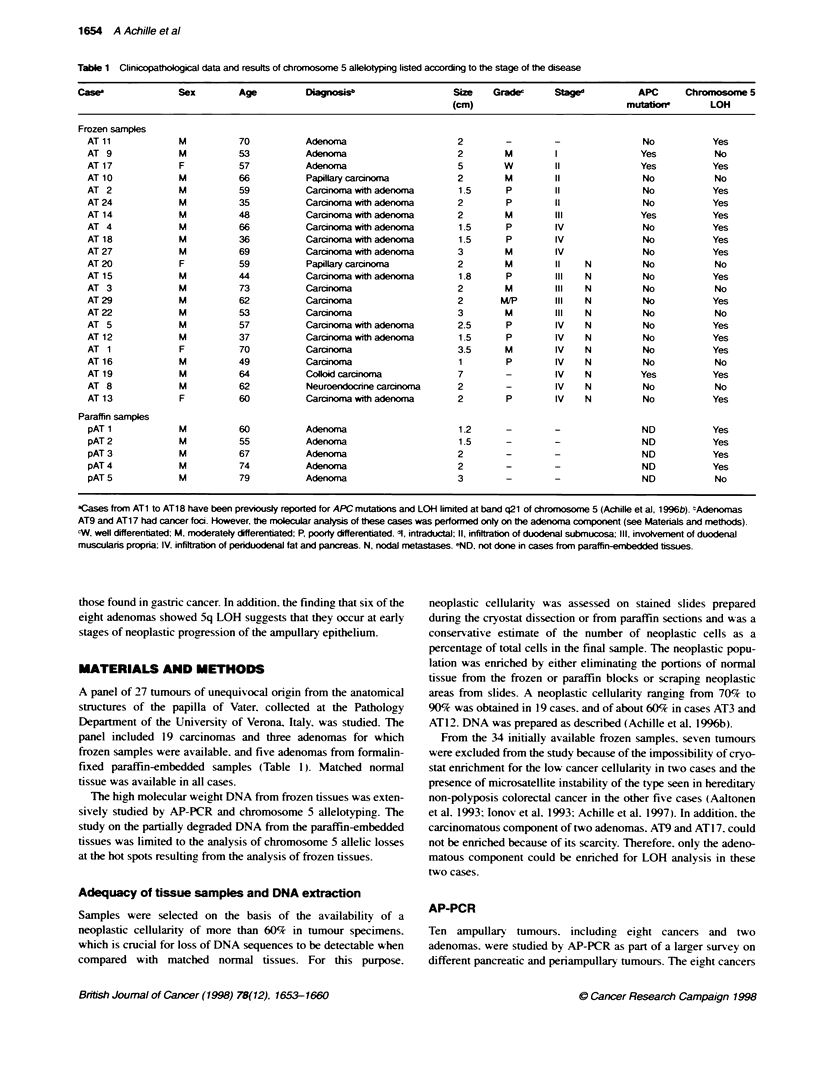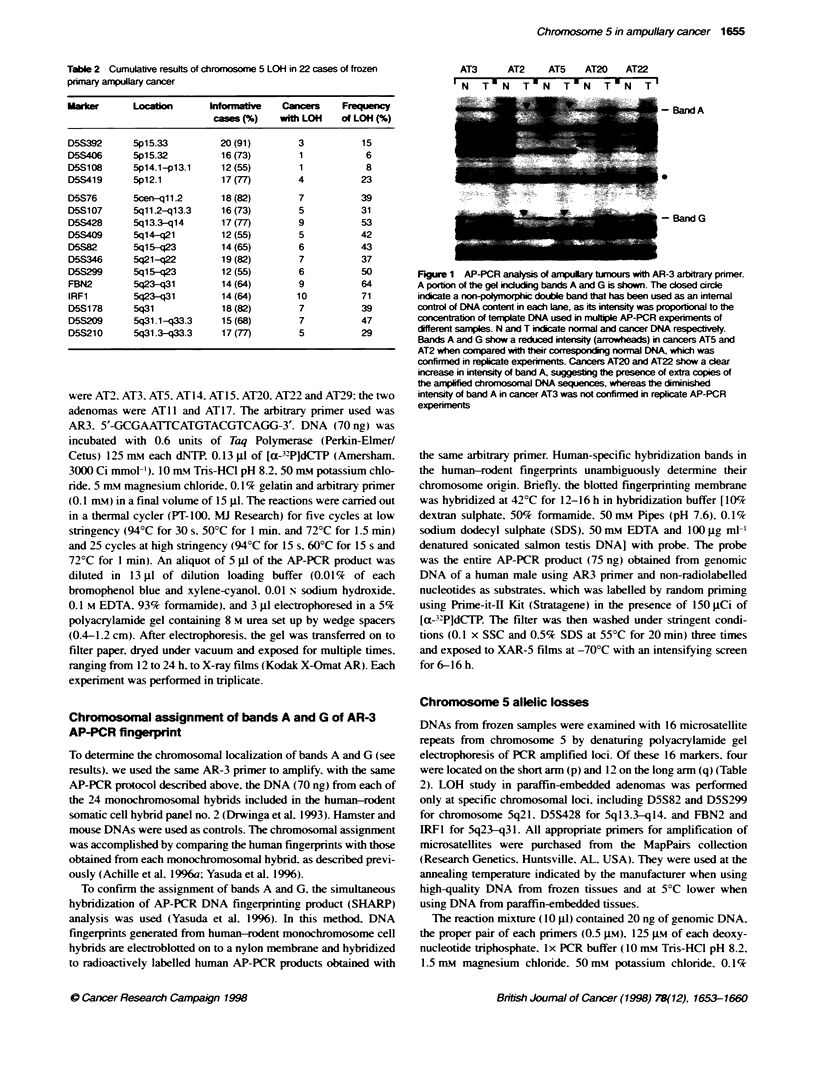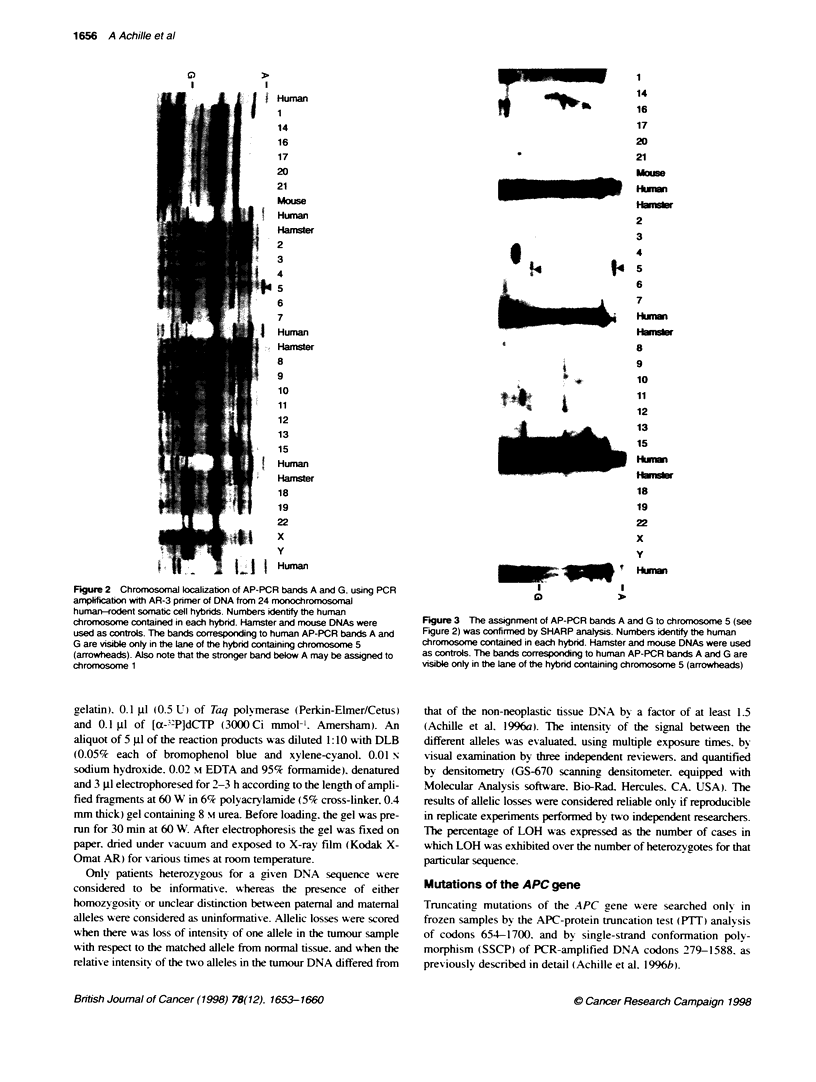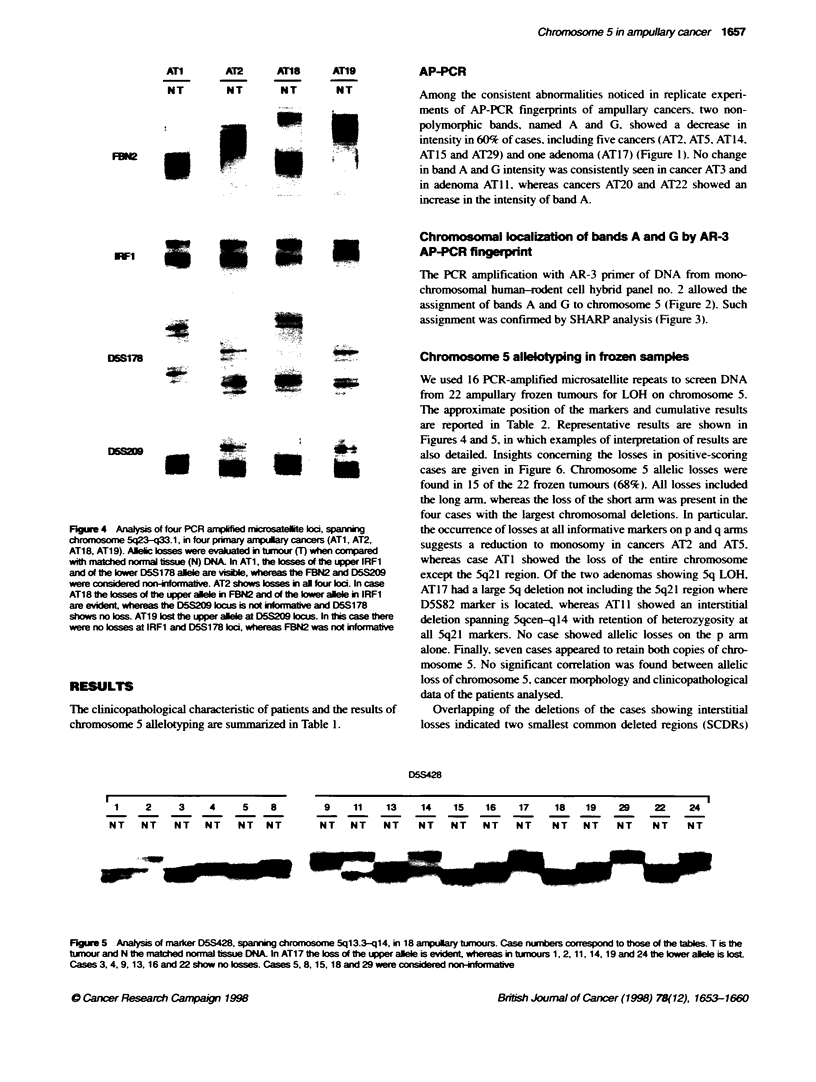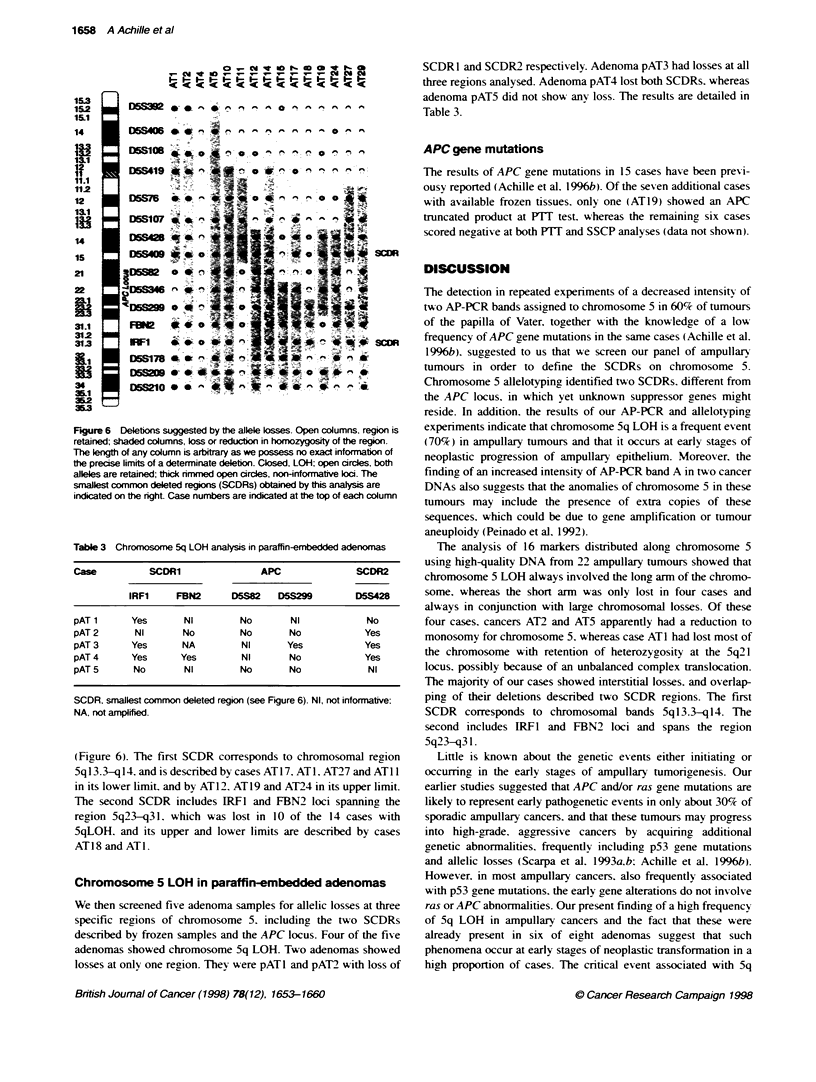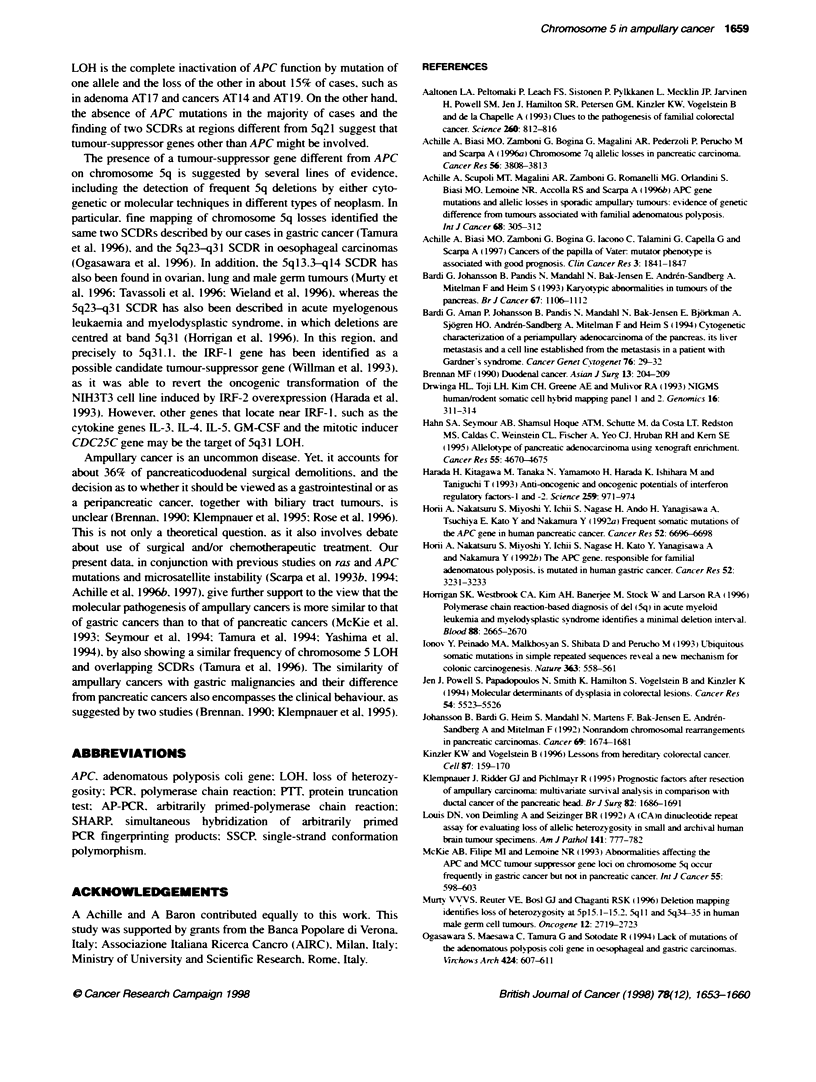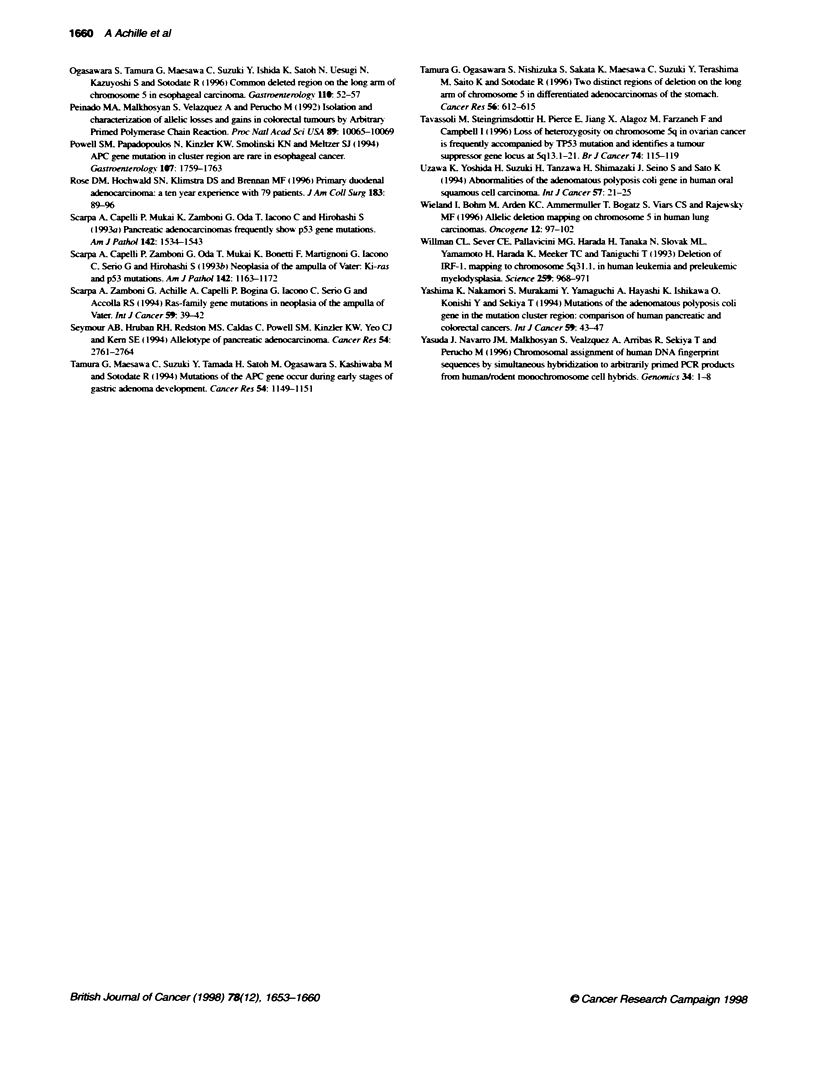# Chromosome 5 allelic losses are early events in tumours of the papilla of Vater and occur at sites similar to those of gastric cancer.

**DOI:** 10.1038/bjc.1998.738

**Published:** 1998-12

**Authors:** A. Achille, A. Baron, G. Zamboni, C. Di Pace, S. Orlandini, A. Scarpa

**Affiliations:** Istituto di Anatomia Patologica, Università di Verona, Italy.

## Abstract

**Images:**


British Journal of Cancer (1998) 78(12), 1653–1660
© 1998 Cancer Research Campaign

### Chromosome 5 allelic losses are early events in tumours of the papilla of Vater and occur at sites similar to those of gastric cancer

**A Achille, A Baron, G Zamboni, C Di Pace, S Orlandini and A Scarpa**

Istituto di Anatomia Patologica, Università di Verona, Strada Le Grazie 8, I-37134 Verona, Italy

**Summary** During our studies of DNA fingerprinting of tumours of the pancreas and papilla (ampulla) of Vater, using arbitrarily primed polymerase chain reaction (AP-PCR), we noticed two bands showing a decreased intensity in six of ten ampullary tumours with respect to matched normal tissues. Those bands were both assigned to chromosome 5. Such a finding was somewhat in contrast with the reportedly low frequency of *APC* gene mutations in ampullary cancers, located at chromosome 5q21, and suggested that loci different from that of *APC* might be the target of chromosome 5 allelic losses (LOH) in these tumours. Therefore, we analysed chromosome 5 LOH in a panel of 27 ampullary tumours, including eight adenomas, four early- and 15 advanced-stage cancers, using 16 PCR-amplified CA microsatellite polymorphic markers spanning the entire chromosome. Nineteen cases (70%) showed LOH, and the interstitial deletions found in these tumours described two smallest common deleted regions, in which putative suppressor genes might reside. They were at 5q13.3–q14 and at 5q23–q31 respectively, which correspond to those found in gastric tumours. In addition, the presence of 5q LOH in six of eight adenomas and in three of four early-stage cancers suggests that such phenomena occur at early stages of neoplastic progression of the ampullary epithelium.

**Keywords:** papilla of Vater; cancer; arbitrarily primed polymerase chain reaction; loss of heterozygosity; microsatellite

It is widely accepted that accumulation of genetic changes underlies the development of cancer. However, there is evidence that it is not simply the accumulation of mutations, but also their order, that determines the propensity for neoplasia, and that only a subset of the genes that can affect cell growth can actually initiate the neoplastic process (Kinzler and Vogelstein, 1996). Progress in understanding the pathogenesis of different malignancies will in large part depend on identifying the early genetic anomalies involved in the initiation of neoplastic transformation. The earliest genetic event in the great majority of colorectal cancers is inactivation of the adenomatosis polyposis coli (*APC*) gene (Jen et al, 1994). However, *APC* gene mutation seems not to be a step-limiting event of neoplastic progression in gastrointestinal cancers other than colorectal. In fact, *APC* mutations are infrequent in oral, oesophageal, gastric, pancreatic and liver cancer, and, with the possible exception of Ki-*ras* mutations in pancreatic cancer, the earliest genetic alteration in these malignancies has not yet been discovered (Horii et al, 1992*a*; *b*; McKie et al, 1993; Ogasawara et al, 1994; Powell et al, 1994; Seymour et al, 1994; Uzawa et al, 1994; Yashima et al, 1994; Hahn et al, 1995).

During our studies of DNA fingerprinting of cancers of the pancreas and of the papilla (ampulla) of Vater, using arbitrarily primed polymerase chain reaction (AP-PCR), we noticed two bands showing a decreased intensity in six of ten ampullary tumours, including one adenoma, with respect to matched normal tissues. It has been demonstrated that decreased intensity of AP-PCR bands in tumour DNA reflects allelic losses, whereas increased band intensity indicates the presence of extra copies of these sequences (Achille et al, 1996*a*; Peinado et al, 1992). The two AP-PCR bands were both assigned to chromosome 5. Such information led us to analyse the entire chromosome 5 for the presence of allelic losses (LOHs) in a panel of ampullary tumours, in addition to the fact that our previous observation of a low frequency of *APC* gene mutations in these cancers (Achille et al, 1996*b*) suggested that the *APC* gene, located at chromosome 5q21, might not be the exclusive or most important target of chromosome 5 deletions in ampullary tumours. In this respect, it is worthy of note that no structural abnormality of chromosome 5 had been reported in the nine ampullary or periampullary carcinomas in which cytogenetic analysis was performed (Johansson et al, 1992; Bardi et al, 1993, 1994). Molecular techniques are more sensitive than cytogenetic analysis in detecting loss of genetic material, wherever it is located, either in its natural position or in the context of complex translocations, and in determining the smallest common deleted regions (SCDRs) involved in those alterations. These methods are based on the detection of LOH at chromosome-specific polymorphic sites in DNA extracted from tumour, when compared with DNA from matched normal tissues. Such 'allelotyping' is feasible by PCR amplification of microsatellite repeats, provided the heterozygosity for the studied loci and neoplastic cellularity are higher than 50–60% in the cancer sample (Louis et al, 1992).

We explored chromosome 5 LOH in 27 ampullary tumours, including eight adenomas, using PCR amplification of matched normal and cancer DNAs with a set of CA microsatellite repeat-specific primers. Our results indicate that chromosome 5q LOH is frequent (70%) in ampullary tumours, and point to the presence of two different SCDRs, away from the *APC* locus and similar to

*Received 23 October 1997*

*Revised 30 March 1998*

*Accepted 7 April 1998*

*Correspondence to:* A Scarpa

**Table 1** Clinicopathological data and results of chromosome 5 allelotyping listed according to the stage of the disease

| Case[a] | Sex | Age | Diagnosis[b] | Size (cm) | Grade[c] | Stage[d] | | APC mutation[e] | Chromosome 5 LOH |
|---|---|---|---|---|---|---|---|---|---|
| Frozen samples | | | | | | | | | |
| AT 11 | M | 70 | Adenoma | 2 | – | – | | No | Yes |
| AT 9 | M | 53 | Adenoma | 2 | M | I | | Yes | No |
| AT 17 | F | 57 | Adenoma | 5 | W | II | | Yes | Yes |
| AT 10 | M | 66 | Papillary carcinoma | 2 | M | II | | No | No |
| AT 2 | M | 59 | Carcinoma with adenoma | 1.5 | P | II | | No | Yes |
| AT 24 | M | 35 | Carcinoma with adenoma | 2 | P | II | | No | Yes |
| AT 14 | M | 48 | Carcinoma with adenoma | 2 | M | III | | Yes | Yes |
| AT 4 | M | 66 | Carcinoma with adenoma | 1.5 | P | IV | | No | Yes |
| AT 18 | M | 36 | Carcinoma with adenoma | 1.5 | P | IV | | No | Yes |
| AT 27 | M | 69 | Carcinoma with adenoma | 3 | M | IV | | No | Yes |
| AT 20 | F | 59 | Papillary carcinoma | 2 | M | II | N | No | No |
| AT 15 | M | 44 | Carcinoma with adenoma | 1.8 | P | III | N | No | Yes |
| AT 3 | M | 73 | Carcinoma | 2 | M | III | N | No | No |
| AT 29 | M | 62 | Carcinoma | 2 | M/P | III | N | No | Yes |
| AT 22 | M | 53 | Carcinoma | 3 | M | III | N | No | No |
| AT 5 | M | 57 | Carcinoma with adenoma | 2.5 | P | IV | N | No | Yes |
| AT 12 | M | 37 | Carcinoma with adenoma | 1.5 | P | IV | N | No | Yes |
| AT 1 | F | 70 | Carcinoma | 3.5 | M | IV | N | No | Yes |
| AT 16 | M | 49 | Carcinoma | 1 | P | IV | N | No | No |
| AT 19 | M | 64 | Colloid carcinoma | 7 | – | IV | N | Yes | Yes |
| AT 8 | M | 62 | Neuroendocrine carcinoma | 2 | – | IV | N | No | No |
| AT 13 | F | 60 | Carcinoma with adenoma | 2 | P | IV | N | No | Yes |
| Paraffin samples | | | | | | | | | |
| pAT 1 | M | 60 | Adenoma | 1.2 | – | – | | ND | Yes |
| pAT 2 | M | 55 | Adenoma | 1.5 | – | – | | ND | Yes |
| pAT 3 | M | 67 | Adenoma | 2 | – | – | | ND | Yes |
| pAT 4 | M | 74 | Adenoma | 2 | – | – | | ND | Yes |
| pAT 5 | M | 79 | Adenoma | 3 | – | – | | ND | No |

[a]Cases from AT1 to AT18 have been previously reported for *APC* mutations and LOH limited at band q21 of chromosome 5 (Achille et al, 1996*b*). [b]Adenomas AT9 and AT17 had cancer foci. However, the molecular analysis of these cases was performed only on the adenoma component (see Materials and methods). [c]W, well differentiated; M, moderately differentiated; P, poorly differentiated. [d]I, intraductal; II, infiltration of duodenal submucosa; III, involvement of duodenal muscularis propria; IV, infiltration of periduodenal fat and pancreas. N, nodal metastases. [e]ND, not done in cases from paraffin-embedded tissues.

those found in gastric cancer. In addition, the finding that six of the eight adenomas showed 5q LOH suggests that they occur at early stages of neoplastic progression of the ampullary epithelium.

### MATERIALS AND METHODS

A panel of 27 tumours of unequivocal origin from the anatomical structures of the papilla of Vater, collected at the Pathology Department of the University of Verona, Italy, was studied. The panel included 19 carcinomas and three adenomas for which frozen samples were available, and five adenomas from formalin-fixed paraffin-embedded samples (Table 1). Matched normal tissue was available in all cases.

The high molecular weight DNA from frozen tissues was extensively studied by AP-PCR and chromosome 5 allelotyping. The study on the partially degraded DNA from the paraffin-embedded tissues was limited to the analysis of chromosome 5 allelic losses at the hot spots resulting from the analysis of frozen tissues.

### Adequacy of tissue samples and DNA extraction

Samples were selected on the basis of the availability of a neoplastic cellularity of more than 60% in tumour specimens, which is crucial for loss of DNA sequences to be detectable when compared with matched normal tissues. For this purpose, neoplastic cellularity was assessed on stained slides prepared during the cryostat dissection or from paraffin sections and was a conservative estimate of the number of neoplastic cells as a percentage of total cells in the final sample. The neoplastic population was enriched by either eliminating the portions of normal tissue from the frozen or paraffin blocks or scraping neoplastic areas from slides. A neoplastic cellularity ranging from 70% to 90% was obtained in 19 cases, and of about 60% in cases AT3 and AT12. DNA was prepared as described (Achille et al, 1996b).

From the 34 initially available frozen samples, seven tumours were excluded from the study because of the impossibility of cryostat enrichment for the low cancer cellularity in two cases and the presence of microsatellite instability of the type seen in hereditary non-polyposis colorectal cancer in the other five cases (Aaltonen et al, 1993; Ionov et al, 1993; Achille et al, 1997). In addition, the carcinomatous component of two adenomas, AT9 and AT17, could not be enriched because of its scarcity. Therefore, only the adenomatous component could be enriched for LOH analysis in these two cases.

### AP-PCR

Ten ampullary tumours, including eight cancers and two adenomas, were studied by AP-PCR as part of a larger survey on different pancreatic and periampullary tumours. The eight cancers

*© Cancer Research Campaign 1998*

Table 2 Cumulative results of chromosome 5 LOH in 22 cases of frozen primary ampullary cancer

| Marker | Location | Informative cases (%) | Cancers with LOH | Frequency of LOH (%) |
|---|---|---|---|---|
| D5S392 | 5p15.33 | 20 (91) | 3 | 15 |
| D5S406 | 5p15.32 | 16 (73) | 1 | 6 |
| D5S108 | 5p14.1–p13.1 | 12 (55) | 1 | 8 |
| D5S419 | 5p12.1 | 17 (77) | 4 | 23 |
| D5S76 | 5cen–q11.2 | 18 (82) | 7 | 39 |
| D5S107 | 5q11.2–q13.3 | 16 (73) | 5 | 31 |
| D5S428 | 5q13.3–q14 | 17 (77) | 9 | 53 |
| D5S409 | 5q14–q21 | 12 (55) | 5 | 42 |
| D5S82 | 5q15–q23 | 14 (65) | 6 | 43 |
| D5S346 | 5q21–q22 | 19 (82) | 7 | 37 |
| D5S299 | 5q15–q23 | 12 (55) | 6 | 50 |
| FBN2 | 5q23–q31 | 14 (64) | 9 | 64 |
| IRF1 | 5q23–q31 | 14 (64) | 10 | 71 |
| D5S178 | 5q31 | 18 (82) | 7 | 39 |
| D5S209 | 5q31.1–q33.3 | 15 (68) | 7 | 47 |
| D5S210 | 5q31.3–q33.3 | 17 (77) | 5 | 29 |

Figure 1 AP-PCR analysis of ampullary tumours with AR-3 arbitrary primer. A portion of the gel including bands A and G is shown. The closed circle indicate a non-polymorphic double band that has been used as an internal control of DNA content in each lane, as its intensity was proportional to the concentration of template DNA used in multiple AP-PCR experiments of different samples. N and T indicate normal and cancer DNA respectively. Bands A and G show a reduced intensity (arrowheads) in cancers AT5 and AT2 when compared with their corresponding normal DNA, which was confirmed in replicate experiments. Cancers AT20 and AT22 show a clear increase in intensity of band A, suggesting the presence of extra copies of the amplified chromosomal DNA sequences, whereas the diminished intensity of band A in cancer AT3 was not confirmed in replicate AP-PCR experiments were AT2, AT3, AT5, AT14, AT15, AT20, AT22 and AT29; the two adenomas were AT11 and AT17. The arbitrary primer used was AR3, 5′-GCGAATTCATGTACGTCAGG-3′. DNA (70 ng) was incubated with 0.6 units of *Taq* Polymerase (Perkin-Elmer/ Cetus) 125 mM each dNTP, 0.13 µl of [α-$^{32}$P]dCTP (Amersham, 3000 Ci mmol$^{-1}$), 10 mM Tris-HCl pH 8.2, 50 mM potassium chloride, 5 mM magnesium chloride, 0.1% gelatin and arbitrary primer (0.1 mM) in a final volume of 15 µl. The reactions were carried out in a thermal cycler (PT-100, MJ Research) for five cycles at low stringency (94°C for 30 s, 50°C for 1 min, and 72°C for 1.5 min) and 25 cycles at high stringency (94°C for 15 s, 60°C for 15 s and 72°C for 1 min). An aliquot of 5 µl of the AP-PCR product was diluted in 13 µl of dilution loading buffer (0.01% of each bromophenol blue and xylene-cyanol, 0.01 N sodium hydroxide, 0.1 M EDTA, 93% formamide), and 3 µl electrophoresed in a 5% polyacrylamide gel containing 8 M urea set up by wedge spacers (0.4–1.2 cm). After electrophoresis, the gel was transferred on to filter paper, dryed under vacuum and exposed for multiple times, ranging from 12 to 24 h, to X-ray films (Kodak X-Omat AR). Each experiment was performed in triplicate.

### Chromosomal assignment of bands A and G of AR-3 AP-PCR fingerprint

To determine the chromosomal localization of bands A and G (see results), we used the same AR-3 primer to amplify, with the same AP-PCR protocol described above, the DNA (70 ng) from each of the 24 monochromosomal hybrids included in the human–rodent somatic cell hybrid panel no. 2 (Drwinga et al, 1993). Hamster and mouse DNAs were used as controls. The chromosomal assignment was accomplished by comparing the human fingerprints with those obtained from each monochromosomal hybrid, as described previously (Achille et al, 1996*a*; Yasuda et al, 1996).

To confirm the assignment of bands A and G, the simultaneous hybridization of AP-PCR DNA fingerprinting product (SHARP) analysis was used (Yasuda et al, 1996). In this method, DNA fingerprints generated from human–rodent monochromosome cell hybrids are electroblotted on to a nylon membrane and hybridized to radioactively labelled human AP-PCR products obtained with the same arbitrary primer. Human-specific hybridization bands in the human–rodent fingerprints unambiguously determine their chromosome origin. Briefly, the blotted fingerprinting membrane was hybridized at 42°C for 12–16 h in hybridization buffer [10% dextran sulphate, 50% formamide, 50 mM Pipes (pH 7.6), 0.1% sodium dodecyl sulphate (SDS), 50 mM EDTA and 100 µg ml$^{-1}$ denatured sonicated salmon testis DNA] with probe. The probe was the entire AP-PCR product (75 ng) obtained from genomic DNA of a human male using AR3 primer and non-radiolabelled nucleotides as substrates, which was labelled by random priming using Prime-it-II Kit (Stratagene) in the presence of 150 µCi of [α-$^{32}$P]dCTP. The filter was then washed under stringent conditions (0.1 × SSC and 0.5% SDS at 55°C for 20 min) three times and exposed to XAR-5 films at –70°C with an intensifying screen for 6–16 h.

### Chromosome 5 allelic losses

DNAs from frozen samples were examined with 16 microsatellite repeats from chromosome 5 by denaturing polyacrylamide gel electrophoresis of PCR amplified loci. Of these 16 markers, four were located on the short arm (p) and 12 on the long arm (q) (Table 2). LOH study in paraffin-embedded adenomas was performed only at specific chromosomal loci, including D5S82 and D5S299 for chromosome 5q21, D5S428 for 5q13.3–q14, and FBN2 and IRF1 for 5q23–q31. All appropriate primers for amplification of microsatellites were purchased from the MapPairs collection (Research Genetics, Huntsville, AL, USA). They were used at the annealing temperature indicated by the manufacturer when using high-quality DNA from frozen tissues and at 5°C lower when using DNA from paraffin-embedded tissues.

The reaction mixture (10 µl) contained 20 ng of genomic DNA, the proper pair of each primers (0.5 µM), 125 µM of each deoxynucleotide triphosphate, 1× PCR buffer (10 mM Tris-HCl pH 8.2, 1.5 mM magnesium chloride, 50 mM potassium chloride, 0.1%

© Cancer Research Campaign 1998 

**Figure 2** Chromosomal localization of AP-PCR bands A and G, using PCR amplification with AR-3 primer of DNA from 24 monochromosomal human–rodent somatic cell hybrids. Numbers identify the human chromosome contained in each hybrid. Hamster and mouse DNAs were used as controls. The bands corresponding to human AP-PCR bands A and G are visible only in the lane of the hybrid containing chromosome 5 (arrowheads). Also note that the stronger band below A may be assigned to chromosome 1

**Figure 3** The assignment of AP-PCR bands A and G to chromosome 5 (see Figure 2) was confirmed by SHARP analysis. Numbers identify the human chromosome contained in each hybrid. Hamster and mouse DNAs were used as controls. The bands corresponding to human AP-PCR bands A and G are visible only in the lane of the hybrid containing chromosome 5 (arrowheads)

gelatin), 0.1 μl (0.5 U) of *Taq* polymerase (Perkin-Elmer/Cetus) and 0.1 μl of [$\alpha$-$^{32}$P]dCTP (3000 Ci mmol$^{-1}$, Amersham). An aliquot of 5 μl of the reaction products was diluted 1:10 with DLB (0.05% each of bromophenol blue and xylene-cyanol, 0.01 N sodium hydroxide, 0.02 M EDTA and 95% formamide), denatured and 3 μl electrophoresed for 2–3 h according to the length of amplified fragments at 60 W in 6% polyacrylamide (5% cross-linker, 0.4 mm thick) gel containing 8 M urea. Before loading, the gel was prerun for 30 min at 60 W. After electrophoresis the gel was fixed on paper, dried under vacuum and exposed to X-ray film (Kodak X-Omat AR) for various times at room temperature.

Only patients heterozygous for a given DNA sequence were considered to be informative, whereas the presence of either homozygosity or unclear distinction between paternal and maternal alleles were considered as uninformative. Allelic losses were scored when there was loss of intensity of one allele in the tumour sample with respect to the matched allele from normal tissue, and when the relative intensity of the two alleles in the tumour DNA differed from that of the non-neoplastic tissue DNA by a factor of at least 1.5 (Achille et al, 1996*a*). The intensity of the signal between the different alleles was evaluated, using multiple exposure times, by visual examination by three independent reviewers, and quantified by densitometry (GS-670 scanning densitometer, equipped with Molecular Analysis software, Bio-Rad, Hercules, CA, USA). The results of allelic losses were considered reliable only if reproducible in replicate experiments performed by two independent researchers. The percentage of LOH was expressed as the number of cases in which LOH was exhibited over the number of heterozygotes for that particular sequence.

### Mutations of the *APC* gene

Truncating mutations of the *APC* gene were searched only in frozen samples by the APC-protein truncation test (PTT) analysis of codons 654–1700, and by single-strand conformation polymorphism (SSCP) of PCR-amplified DNA codons 279–1588, as previously described in detail (Achille et al, 1996*b*).

© Cancer Research Campaign 1998

**Figure 4** Analysis of four PCR amplified microsatellite loci, spanning chromosome 5q23–q33.1, in four primary ampullary cancers (AT1, AT2, AT18, AT19). Allelic losses were evaluated in tumour (T) when compared with matched normal tissue (N) DNA. In AT1, the losses of the upper IRF1 and of the lower D5S178 allele are visible, whereas the FBN2 and D5S209 were considered non-informative. AT2 shows losses in all four loci. In case AT18 the losses of the upper allele in FBN2 and of the lower allele in IRF1 are evident, whereas the D5S209 locus is not informative and D5S178 shows no loss. AT19 lost the upper allele at D5S209 locus. In this case there were no losses at IRF1 and D5S178 loci, whereas FBN2 was not informative

### RESULTS

The clinicopathological characteristic of patients and the results of chromosome 5 allelotyping are summarized in Table 1.

### AP-PCR

Among the consistent abnormalities noticed in replicate experiments of AP-PCR fingerprints of ampullary cancers, two non-polymorphic bands, named A and G, showed a decrease in intensity in 60% of cases, including five cancers (AT2, AT5, AT14, AT15 and AT29) and one adenoma (AT17) (Figure 1). No change in band A and G intensity was consistently seen in cancer AT3 and in adenoma AT11, whereas cancers AT20 and AT22 showed an increase in the intensity of band A.

### Chromosomal localization of bands A and G by AR-3 AP-PCR fingerprint

The PCR amplification with AR-3 primer of DNA from monochromosomal human–rodent cell hybrid panel no. 2 allowed the assignment of bands A and G to chromosome 5 (Figure 2). Such assignment was confirmed by SHARP analysis (Figure 3).

### Chromosome 5 allelotyping in frozen samples

We used 16 PCR-amplified microsatellite repeats to screen DNA from 22 ampullary frozen tumours for LOH on chromosome 5. The approximate position of the markers and cumulative results are reported in Table 2. Representative results are shown in Figures 4 and 5, in which examples of interpretation of results are also detailed. Insights concerning the losses in positive-scoring cases are given in Figure 6. Chromosome 5 allelic losses were found in 15 of the 22 frozen tumours (68%). All losses included the long arm, whereas the loss of the short arm was present in the four cases with the largest chromosomal deletions. In particular, the occurrence of losses at all informative markers on p and q arms suggests a reduction to monosomy in cancers AT2 and AT5, whereas case AT1 showed the loss of the entire chromosome except the 5q21 region. Of the two adenomas showing 5q LOH, AT17 had a large 5q deletion not including the 5q21 region where D5S82 marker is located, whereas AT11 showed an interstitial deletion spanning 5qcen–q14 with retention of heterozygosity at all 5q21 markers. No case showed allelic losses on the p arm alone. Finally, seven cases appeared to retain both copies of chromosome 5. No significant correlation was found between allelic loss of chromosome 5, cancer morphology and clinicopathological data of the patients analysed.

Overlapping of the deletions of the cases showing interstitial losses indicated two smallest common deleted regions (SCDRs)

**Figure 5** Analysis of marker D5S428, spanning chromosome 5q13.3–q14, in 18 ampullary tumours. Case numbers correspond to those of the tables. T is the tumour and N the matched normal tissue DNA. In AT17 the loss of the upper allele is evident, whereas in tumours 1, 2, 11, 14, 19 and 24 the lower allele is lost. Cases 3, 4, 9, 13, 16 and 22 show no losses. Cases 5, 8, 15, 18 and 29 were considered non-informative

**Figure 6** Deletions suggested by the allele losses. Open columns, region is retained; shaded columns, loss or reduction in homozygosity of the region. The length of any column is arbitrary as we possess no exact information of the precise limits of a determinate deletion. Closed, LOH; open circles, both alleles are retained; thick rimmed open circles, non-informative loci. The smallest common deleted regions (SCDRs) obtained by this analysis are indicated on the right. Case numbers are indicated at the top of each column

**Table 3** Chromosome 5q LOH analysis in paraffin-embedded adenomas

| Case | SCDR1 | | APC | | SCDR2 |
|---|---|---|---|---|---|
| | IRF1 | FBN2 | D5S82 | D5S299 | D5S428 |
| pAT 1 | Yes | NI | No | NI | No |
| pAT 2 | NI | No | No | No | Yes |
| pAT 3 | Yes | NA | NI | Yes | Yes |
| pAT 4 | Yes | Yes | NI | No | Yes |
| pAT 5 | No | NI | No | No | NI |

SCDR, smallest common deleted region (see Figure 6). NI, not informative; NA, not amplified.

(Figure 6). The first SCDR corresponds to chromosomal region 5q13.3–q14, and is described by cases AT17, AT1, AT27 and AT11 in its lower limit, and by AT12, AT19 and AT24 in its upper limit. The second SCDR includes IRF1 and FBN2 loci spanning the region 5q23–q31, which was lost in 10 of the 14 cases with 5qLOH, and its upper and lower limits are described by cases AT18 and AT1.

### Chromosome 5 LOH in paraffin-embedded adenomas

We then screened five adenoma samples for allelic losses at three specific regions of chromosome 5, including the two SCDRs described by frozen samples and the *APC* locus. Four of the five adenomas showed chromosome 5q LOH. Two adenomas showed losses at only one region. They were pAT1 and pAT2 with loss of SCDR1 and SCDR2 respectively. Adenoma pAT3 had losses at all three regions analysed. Adenoma pAT4 lost both SCDRs, whereas adenoma pAT5 did not show any loss. The results are detailed in Table 3.

### *APC* gene mutations

The results of *APC* gene mutations in 15 cases have been previousy reported (Achille et al, 1996*b*). Of the seven additional cases with available frozen tissues, only one (AT19) showed an APC truncated product at PTT test, whereas the remaining six cases scored negative at both PTT and SSCP analyses (data not shown).

### DISCUSSION

The detection in repeated experiments of a decreased intensity of two AP-PCR bands assigned to chromosome 5 in 60% of tumours of the papilla of Vater, together with the knowledge of a low frequency of *APC* gene mutations in the same cases (Achille et al, 1996*b*), suggested to us that we screen our panel of ampullary tumours in order to define the SCDRs on chromosome 5. Chromosome 5 allelotyping identified two SCDRs, different from the *APC* locus, in which yet unknown suppressor genes might reside. In addition, the results of our AP-PCR and allelotyping experiments indicate that chromosome 5q LOH is a frequent event (70%) in ampullary tumours and that it occurs at early stages of neoplastic progression of ampullary epithelium. Moreover, the finding of an increased intensity of AP-PCR band A in two cancer DNAs also suggests that the anomalies of chromosome 5 in these tumours may include the presence of extra copies of these sequences, which could be due to gene amplification or tumour aneuploidy (Peinado et al, 1992).

The analysis of 16 markers distributed along chromosome 5 using high-quality DNA from 22 ampullary tumours showed that chromosome 5 LOH always involved the long arm of the chromosome, whereas the short arm was only lost in four cases and always in conjunction with large chromosomal losses. Of these four cases, cancers AT2 and AT5 apparently had a reduction to monosomy for chromosome 5, whereas case AT1 had lost most of the chromosome with retention of heterozygosity at the 5q21 locus, possibly because of an unbalanced complex translocation. The majority of our cases showed interstitial losses, and overlapping of their deletions described two SCDR regions. The first SCDR corresponds to chromosomal bands 5q13.3–q14. The second includes IRF1 and FBN2 loci and spans the region 5q23–q31.

Little is known about the genetic events either initiating or occurring in the early stages of ampullary tumorigenesis. Our earlier studies suggested that *APC* and/or *ras* gene mutations are likely to represent early pathogenetic events in only about 30% of sporadic ampullary cancers, and that these tumours may progress into high-grade, aggressive cancers by acquiring additional genetic abnormalities, frequently including p53 gene mutations and allelic losses (Scarpa et al, 1993*a*,*b*; Achille et al, 1996*b*). However, in most ampullary cancers, also frequently associated with p53 gene mutations, the early gene alterations do not involve *ras* or *APC* abnormalities. Our present finding of a high frequency of 5q LOH in ampullary cancers and the fact that these were already present in six of eight adenomas suggest that such phenomena occur at early stages of neoplastic transformation in a high proportion of cases. The critical event associated with 5q

 © Cancer Research Campaign 1998

LOH is the complete inactivation of *APC* function by mutation of one allele and the loss of the other in about 15% of cases, such as in adenoma AT17 and cancers AT14 and AT19. On the other hand, the absence of *APC* mutations in the majority of cases and the finding of two SCDRs at regions different from 5q21 suggest that tumour-suppressor genes other than *APC* might be involved.

The presence of a tumour-suppressor gene different from *APC* on chromosome 5q is suggested by several lines of evidence, including the detection of frequent 5q deletions by either cytogenetic or molecular techniques in different types of neoplasm. In particular, fine mapping of chromosome 5q losses identified the same two SCDRs described by our cases in gastric cancer (Tamura et al, 1996), and the 5q23–q31 SCDR in oesophageal carcinomas (Ogasawara et al, 1996). In addition, the 5q13.3–q14 SCDR has also been found in ovarian, lung and male germ tumours (Murty et al, 1996; Tavassoli et al, 1996; Wieland et al, 1996), whereas the 5q23–q31 SCDR has also been described in acute myelogenous leukaemia and myelodysplastic syndrome, in which deletions are centred at band 5q31 (Horrigan et al, 1996). In this region, and precisely to 5q31.1, the IRF-1 gene has been identified as a possible candidate tumour-suppressor gene (Willman et al, 1993), as it was able to revert the oncogenic transformation of the NIH3T3 cell line induced by IRF-2 overexpression (Harada et al, 1993). However, other genes that locate near IRF-1, such as the cytokine genes IL-3, IL-4, IL-5, GM-CSF and the mitotic inducer *CDC25C* gene may be the target of 5q31 LOH.

Ampullary cancer is an uncommon disease. Yet, it accounts for about 36% of pancreaticoduodenal surgical demolitions, and the decision as to whether it should be viewed as a gastrointestinal or as a peripancreatic cancer, together with biliary tract tumours, is unclear (Brennan, 1990; Klempnauer et al, 1995; Rose et al, 1996). This is not only a theoretical question, as it also involves debate about use of surgical and/or chemotherapeutic treatment. Our present data, in conjunction with previous studies on *ras* and *APC* mutations and microsatellite instability (Scarpa et al, 1993*b*, 1994; Achille et al, 1996*b*, 1997), give further support to the view that the molecular pathogenesis of ampullary cancers is more similar to that of gastric cancers than to that of pancreatic cancers (McKie et al, 1993; Seymour et al, 1994; Tamura et al, 1994; Yashima et al, 1994), by also showing a similar frequency of chromosome 5 LOH and overlapping SCDRs (Tamura et al, 1996). The similarity of ampullary cancers with gastric malignancies and their difference from pancreatic cancers also encompasses the clinical behaviour, as suggested by two studies (Brennan, 1990; Klempnauer et al, 1995).

### ABBREVIATIONS

*APC*, adenomatous polyposis coli gene; LOH, loss of heterozygosity; PCR, polymerase chain reaction; PTT, protein truncation test; AP-PCR, arbitrarily primed-polymerase chain reaction; SHARP, simultaneous hybridization of arbitrarily primed PCR fingerprinting products; SSCP, single-strand conformation polymorphism.

### ACKNOWLEDGEMENTS

A Achille and A Baron contributed equally to this work. This study was supported by grants from the Banca Popolare di Verona, Italy; Associazione Italiana Ricerca Cancro (AIRC), Milan, Italy; Ministry of University and Scientific Research, Rome, Italy.

### REFERENCES

Aaltonen LA, Peltomaki P, Leach FS, Sistonen P, Pylkkanen L, Mecklin JP, Jarvinen H, Powell SM, Jen J, Hamilton SR, Petersen GM, Kinzler KW, Vogelstein B and de la Chapelle A (1993) Clues to the pathogenesis of familial colorectal cancer. *Science* **260**: 812–816

Achille A, Biasi MO, Zamboni G, Bogina G, Magalini AR, Pederzoli P, Perucho M and Scarpa A (1996*a*) Chromosome 7q allelic losses in pancreatic carcinoma. *Cancer Res* **56**: 3808–3813

Achille A, Scupoli MT, Magalini AR, Zamboni G, Romanelli MG, Orlandini S, Biasi MO, Lemoine NR, Accolla RS and Scarpa A (1996*b*) APC gene mutations and allelic losses in sporadic ampullary tumours: evidence of genetic difference from tumours associated with familial adenomatous polyposis. *Int J Cancer* **68**: 305–312

Achille A, Biasi MO, Zamboni G, Bogina G, Iacono C, Talamini G, Capella G and Scarpa A (1997) Cancers of the papilla of Vater: mutator phenotype is associated with good prognosis. *Clin Cancer Res* **3**: 1841–1847

Bardi G, Johansson B, Pandis N, Mandahl N, Bak-Jensen E, Andrén-Sandberg A, Mitelman F and Heim S (1993) Karyotypic abnormalities in tumours of the pancreas. *Br J Cancer* **67**: 1106–1112

Bardi G, Aman P, Johansson B, Pandis N, Mandahl N, Bak-Jensen E, Björkman A, Sjögren HO, Andrén-Sandberg A, Mitelman F and Heim S (1994) Cytogenetic characterization of a periampullary adenocarcinoma of the pancreas, its liver metastasis and a cell line established from the metastasis in a patient with Gardner's syndrome. *Cancer Genet Cytogenet* **76**: 29–32

Brennan MF (1990) Duodenal cancer. *Asian J Surg* **13**: 204–209

Drwinga HL, Toji LH, Kim CH, Greene AE and Mulivor RA (1993) NIGMS human/rodent somatic cell hybrid mapping panel 1 and 2. *Genomics* **16**: 311–314

Hahn SA, Seymour AB, Shamsul Hoque ATM, Schutte M, da Costa LT, Redston MS, Caldas C, Weinstein CL, Fischer A, Yeo CJ, Hruban RH and Kern SE (1995) Allelotype of pancreatic adenocarcinoma using xenograft enrichment. *Cancer Res* **55**: 4670–4675

Harada H, Kitagawa M, Tanaka N, Yamamoto H, Harada K, Ishihara M and Taniguchi T (1993) Anti-oncogenic and oncogenic potentials of interferon regulatory factors-1 and -2. *Science* **259**: 971–974

Horii A, Nakatsuru S, Miyoshi Y, Ichii S, Nagase H, Ando H, Yanagisawa A, Tsuchiya E, Kato Y and Nakamura Y (1992*a*) Frequent somatic mutations of the *APC* gene in human pancreatic cancer. *Cancer Res* **52**: 6696–6698

Horii A, Nakatsuru S, Miyoshi Y, Ichii S, Nagase H, Kato Y, Yanagisawa A and Nakamura Y (1992*b*) The APC gene, responsible for familial adenomatous polyposis, is mutated in human gastric cancer. *Cancer Res* **52**: 3231–3233

Horrigan SK, Westbrook CA, Kim AH, Banerjee M, Stock W and Larson RA (1996) Polymerase chain reaction-based diagnosis of del (5q) in acute myeloid leukemia and myelodysplastic syndrome identifies a minimal deletion interval. *Blood* **88**: 2665–2670

Ionov Y, Peinado MA, Malkhosyan S, Shibata D and Perucho M (1993) Ubiquitous somatic mutations in simple repeated sequences reveal a new mechanism for colonic carcinogenesis. *Nature* **363**: 558–561

Jen J, Powell S, Papadopoulos N, Smith K, Hamilton S, Vogelstein B and Kinzler K (1994) Molecular determinants of dysplasia in colorectal lesions. *Cancer Res* **54**: 5523–5526

Johansson B, Bardi G, Heim S, Mandahl N, Martens F, Bak-Jensen E, Andrén-Sandberg A and Mitelman F (1992) Nonrandom chromosomal rearrangements in pancreatic carcinomas. *Cancer* **69**: 1674–1681

Kinzler KW and Vogelstein B (1996) Lessons from hereditary colorectal cancer. *Cell* **87**: 159–170

Klempnauer J, Ridder GJ and Pichlmayr R (1995) Prognostic factors after resection of ampullary carcinoma: multivariate survival analysis in comparison with ductal cancer of the pancreatic head. *Br J Surg* **82**: 1686–1691

Louis DN, von Deimling A and Seizinger BR (1992) A (CA)n dinucleotide repeat assay for evaluating loss of allelic heterozygosity in small and archival human brain tumour specimens. *Am J Pathol* **141**: 777–782

McKie AB, Filipe MI and Lemoine NR (1993) Abnormalities affecting the APC and MCC tumour suppressor gene loci on chromosome 5q occur frequently in gastric cancer but not in pancreatic cancer. *Int J Cancer* **55**: 598–603

Murty VVVS, Reuter VE, Bosl GJ and Chaganti RSK (1996) Deletion mapping identifies loss of heterozygosity at 5p15.1–15.2, 5q11 and 5q34–35 in human male germ cell tumours. *Oncogene* **12**: 2719–2723

Ogasawara S, Maesawa C, Tamura G and Sotodate R (1994) Lack of mutations of the adenomatous polyposis coli gene in oesophageal and gastric carcinomas. *Virchows Arch* **424**: 607–611

© Cancer Research Campaign 1998

Ogasawara S. Tamura G. Maesawa C. Suzuki Y. Ishida K. Satoh N. Uesugi N. Kazuyoshi S and Sotodate R (1996) Common deleted region on the long arm of chromosome 5 in esophageal carcinoma. *Gastroenterology* **110**: 52–57

Peinado MA. Malkhosyan S. Velazquez A and Perucho M (1992) Isolation and characterization of allelic losses and gains in colorectal tumours by Arbitrary Primed Polymerase Chain Reaction. *Proc Natl Acad Sci USA* **89**: 10065–10069

Powell SM. Papadopoulos N. Kinzler KW. Smolinski KN and Meltzer SJ (1994) APC gene mutation in cluster region are rare in esophageal cancer. *Gastroenterology* **107**: 1759–1763

Rose DM. Hochwald SN. Klimstra DS and Brennan MF (1996) Primary duodenal adenocarcinoma: a ten year experience with 79 patients. *J Am Coll Surg* **183**: 89–96

Scarpa A. Capelli P. Mukai K. Zamboni G. Oda T. Iacono C and Hirohashi S (1993*a*) Pancreatic adenocarcinomas frequently show p53 gene mutations. *Am J Pathol* **142**: 1534–1543

Scarpa A. Capelli P. Zamboni G. Oda T. Mukai K. Bonetti F. Martignoni G. Iacono C. Serio G and Hirohashi S (1993*b*) Neoplasia of the ampulla of Vater: Ki-*ras* and p53 mutations. *Am J Pathol* **142**: 1163–1172

Scarpa A. Zamboni G. Achille A. Capelli P. Bogina G. Iacono C. Serio G and Accolla RS (1994) Ras-family gene mutations in neoplasia of the ampulla of Vater. *Int J Cancer* **59**: 39–42

Seymour AB. Hruban RH. Redston MS. Caldas C. Powell SM. Kinzler KW. Yeo CJ and Kern SE (1994) Allelotype of pancreatic adenocarcinoma. *Cancer Res* **54**: 2761–2764

Tamura G. Maesawa C. Suzuki Y. Tamada H. Satoh M. Ogasawara S. Kashiwaba M and Sotodate R (1994) Mutations of the APC gene occur during early stages of gastric adenoma development. *Cancer Res* **54**: 1149–1151

Tamura G. Ogasawara S. Nishizuka S. Sakata K. Maesawa C. Suzuki Y. Terashima M. Saito K and Sotodate R (1996) Two distinct regions of deletion on the long arm of chromosome 5 in differentiated adenocarcinomas of the stomach. *Cancer Res* **56**: 612–615

Tavassoli M. Steingrimsdottir H. Pierce E. Jiang X. Alagoz M. Farzaneh F and Campbell I (1996) Loss of heterozygosity on chromosome 5q in ovarian cancer is frequently accompanied by TP53 mutation and identifies a tumour suppressor gene locus at 5q13.1–21. *Br J Cancer* **74**: 115–119

Uzawa K. Yoshida H. Suzuki H. Tanzawa H. Shimazaki J. Seino S and Sato K (1994) Abnormalities of the adenomatous polyposis coli gene in human oral squamous cell carcinoma. *Int J Cancer* **57**: 21–25

Wieland I. Bohm M. Arden KC. Ammermuller T. Bogatz S. Viars CS and Rajewsky MF (1996) Allelic deletion mapping on chromosome 5 in human lung carcinomas. *Oncogene* **12**: 97–102

Willman CL. Sever CE. Pallavicini MG. Harada H. Tanaka N. Slovak ML. Yamamoto H. Harada K. Meeker TC and Taniguchi T (1993) Deletion of IRF-1. mapping to chromosome 5q31.1. in human leukemia and preleukemic myelodysplasia. *Science* **259**: 968–971

Yashima K. Nakamori S. Murakami Y. Yamaguchi A. Hayashi K. Ishikawa O. Konishi Y and Sekiya T (1994) Mutations of the adenomatous polyposis coli gene in the mutation cluster region: comparison of human pancreatic and colorectal cancers. *Int J Cancer* **59**: 43–47

Yasuda J. Navarro JM. Malkhosyan S. Vealzquez A. Arribas R. Sekiya T and Perucho M (1996) Chromosomal assignment of human DNA fingerprint sequences by simultaneous hybridization to arbitrarily primed PCR products from human/rodent monochromosome cell hybrids. *Genomics* **34**: 1–8

*© Cancer Research Campaign 1998*